# Predicting acute pulmonary embolism in COVID-19

**DOI:** 10.1097/MD.0000000000034916

**Published:** 2023-08-25

**Authors:** Ibrahim Koc, Olgun Deniz, Sevda Unalli Ozmen, Sertan Bulut

**Affiliations:** a Bursa City Hospital Pulmonary Medicine, Bursa, Turkey; b Bursa City Hospital, Palliative Care Unit, Geriatric Medicine Clinic, Bursa, Turkey; c Bursa City Hospital Medical Biochemistry, Bursa, Turkey; d Ankara Atatürk Sanatoryum Educational Research Hospital, Bursa, Turkey.

**Keywords:** COVID-19, acute pulmonary embolism, C-reactive protein lymphocyte ratio

## Abstract

Acute pulmonary embolism (PE) is a life-threatening condition in patients with Coronavirus disease-2019 (COVID-19). Computed tomography pulmonary angiography is the preferred test to confirm the diagnosis. However, computed tomography pulmonary angiography is expensive and is not available in every clinic. This study aimed to determine whether clinical findings, symptoms, and parameters that are cost-effective and available in many clinics such as C-reactive protein (CRP) lymphocyte ratio (CLR), and ferritin CRP ratio (FCR) can be used in the diagnosis of PE in patients with COVID-19. Out of the reviewed files, 127 patients were diagnosed with PE, whereas 105 patients had no PE. At the first admission, laboratory parameters, complaints, respiratory rate, and percent oxygen saturation in the blood (SpO2) with a pulse oximeter were recorded for each patient. Eosinophil levels remained lower, whereas ferritin lymphocyte ratio and CLR were higher in the PE group. Patients with more elevated ferritin, CRP, and CLR had an increased mortality risk. Shortness of breath and tiredness was more common in the PE group. A decrease in eosinophil levels, whereas an increase in CLR, D-dimer, and CRP may predict PE. Elevated CLR is highly predictive of PE and is associated with increased mortality risk. COVID-19 patients with a CLR level above 81 should be investigated for PE.

## 1. Introduction

Coronavirus disease-2019 (COVID-19), caused by the severe acute respiratory syndrome-coronavirus 2 (SARS-CoV-2), has been a cause of illness and death of millions since its identification in December 2019. Acute pulmonary embolism (PE) and thromboembolic complications are essential sequelae that contribute to significant morbidity and mortality in patients with COVID-19.^[1,2]^ Previous studies reported elevated inflammatory markers in asthma^[3]^ and excessive inflammation, hypoxia, immobilization, and diffuse intravascular coagulation in the setting of COVID-19 infection as contributors to a prothrombotic state.^[4]^ Evaluation for PE may be challenging because symptoms and some laboratory results of PE overlap with COVID-19. Imaging studies may not be feasible in all cases. The first step to diagnosis is suspicion that the appropriate imaging method is performed if the patient clinical and laboratory findings are compatible. In most cases, Computed tomography with pulmonary angiography (CTPA) is the preferred test to confirm or exclude the diagnosis. However, CTPA is expensive and not available in every clinic, and is radioactive. More straightforward and cheaper methods are needed to help diagnose and assist doctors on which patients must undergo CTPA scanning. Hemogram parameters such as eosinophil levels, monocyte levels, neutrophil-lymphocyte ratio (NLR), and platelet lymphocyte ratio (PLR) have been investigated for diagnosis, prognosis, and severity of COVID-19 patients.^[5,6]^ The purpose of this study was to determine whether clinical findings, complaints, eosinophil levels, C-reactive protein (CRP), lymphocyte ratio (CLR), ferritin lymphocyte ratio (FLR), and ferritin CRP ratio (FCR) can be used in the diagnosis of PE in patients with COVID-19. Also, explore the most useful diagnostic biomarkers and optimal cutoff values, and estimate risk factors for mortality.

## 2. Materials and methods

**Figure 1.**
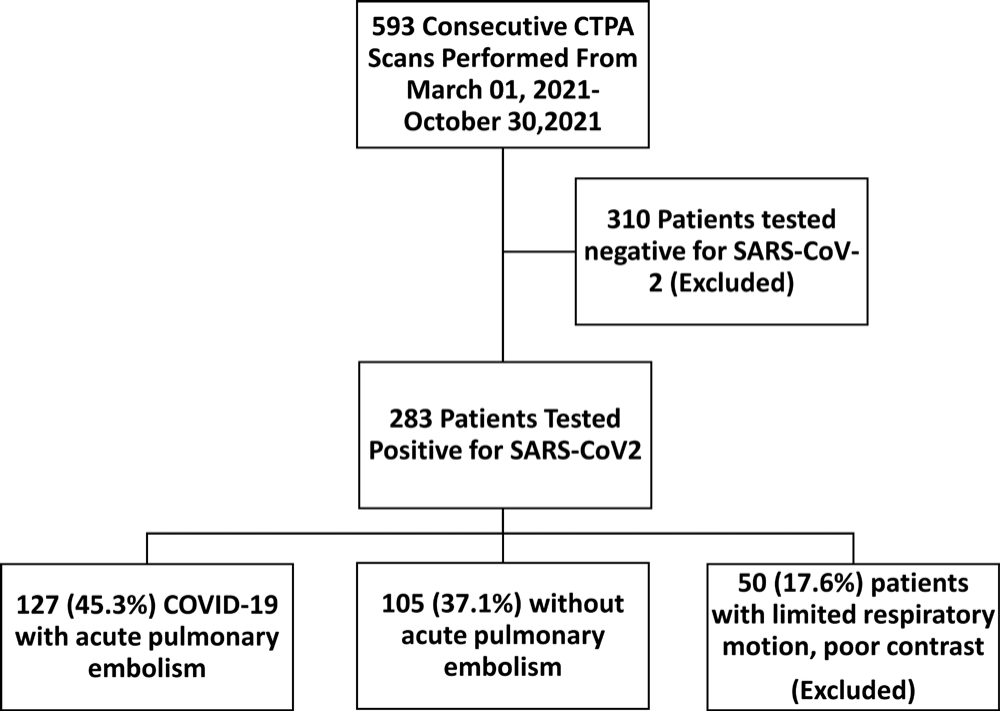
Flowchart of Coronavirus disease-2019 (COVID-19) patients underwent a computed tomography with pulmonary angiography (CTPA) scan.

Files of patients admitted to a tertiary city hospital between March-October 2021 with complaints compatible with COVID-19 were investigated. Five hundred and ninety-three patients underwent a CTPA scanning; out of them, 283 patients tested positive, and 310 tested negative for SARS-CoV-2. Out of those who tested positive, 127 (45.3%) patients had PE, whereas 105 (37.1%) had no PE on CTPA (Fig. 1). Treatment strategies regarding COVID-19 were similar for both groups. Treatment was arranged according to the Ministry of Health COVID-19 Diagnosis and Treatment Guidelines. Since the D-dimer value is elevated in both pulmonary embolism and COVID-19, it may cause confusion. This study aimed to guide physicians about under which conditions CTPA should be performed in cases with a diagnosis of COVID-19 and suspected pulmonary embolism. Therefore, those patients who were referred to pulmonary diseases and underwent CTPA were included in the study. Fifty patients (17.6%) with limited respiratory motion, poor contrast, and patients with immunosuppression and malignancy, which are most likely to affect the blood parameters studied, were excluded from the study. Two patient groups were compared by examining the laboratory parameters obtained on the same admission day before the medical treatment was started in the hospital. Complaints, respiratory rate, and percent oxygen saturation in the blood (SpO2) with a pulse oximeter were recorded for each patient at the first admission. CTPA, polymerized chain reaction testing, and consultation with the relevant specialist were performed during their initial admission to the COVID-19 emergency department, COVID-19 outpatient clinics, and pulmonary medicine outpatient clinics. Severe disease is defined as suspected respiratory infection symptoms in addition to any of the following indicators: shortness of breath, respiratory rate above 30 breaths/min, oxygen saturation at rest below 93%, and PaO2/FiO2 level below 300 mm Hg. CTPA was performed upon the recommendation of a pulmonologist in patients with unexplained shortness of breath or desaturation that cannot be explained by physical examination findings, chest X-ray, or high levels of D-dimer. CRP and D-dimer were examined using the particle-enhanced immunoturbidometric essay. Ferritin levels were assayed using the sandwich principle. Leukocytes were measured using fluorescent flow cytometry; erythrocytes and platelets were measured using the impedance method. Ferritin D-dimer ratio (FDR), FCR, FLR, and ferritin neutrophil ratio values were calculated by dividing ferritin levels by D-dimer, CRP, lymphocyte, and neutrophil count, respectively. Platelet count neutrophil ratio and CLR values were calculated by dividing platelets by neutrophil and CRP to lymphocyte levels. While calculating the ratios, percentage values of blood parameters were not used but absolute blood values were used. The ethics committee approval was obtained from a tertiary hospital Clinical Research Ethics Committee (Ethics Committee Approval No: 2021-16/1). All the statistical analyses were carried out using SPSS 25.0 software. A Kolmogorov–Smirnov test was performed for the normality of the sample data, and the continuous variables were defined by the mean ± standard deviation, and median (minimum-maximum). In contrast, the categorical variables were expressed as frequency and percent. A Student *t* test or a Mann–Whitney *U* test compared the independent groups. The Roc analysis was performed for optimal cutoff values to predict pulmonary embolism. Youden Index was exploited to identify the optimal cutoff values. In addition, a *P* value < .05 was set as the statistical significance level. Spearman correlation coefficient (r) was used to analyze associations between investigated parameters. In all instances, *P* values < .05 were taken to indicate statistical significance. Binary logistic regression analysis was carried out to estimate the mortality risk.

## 3. Results

**Figure 2.**
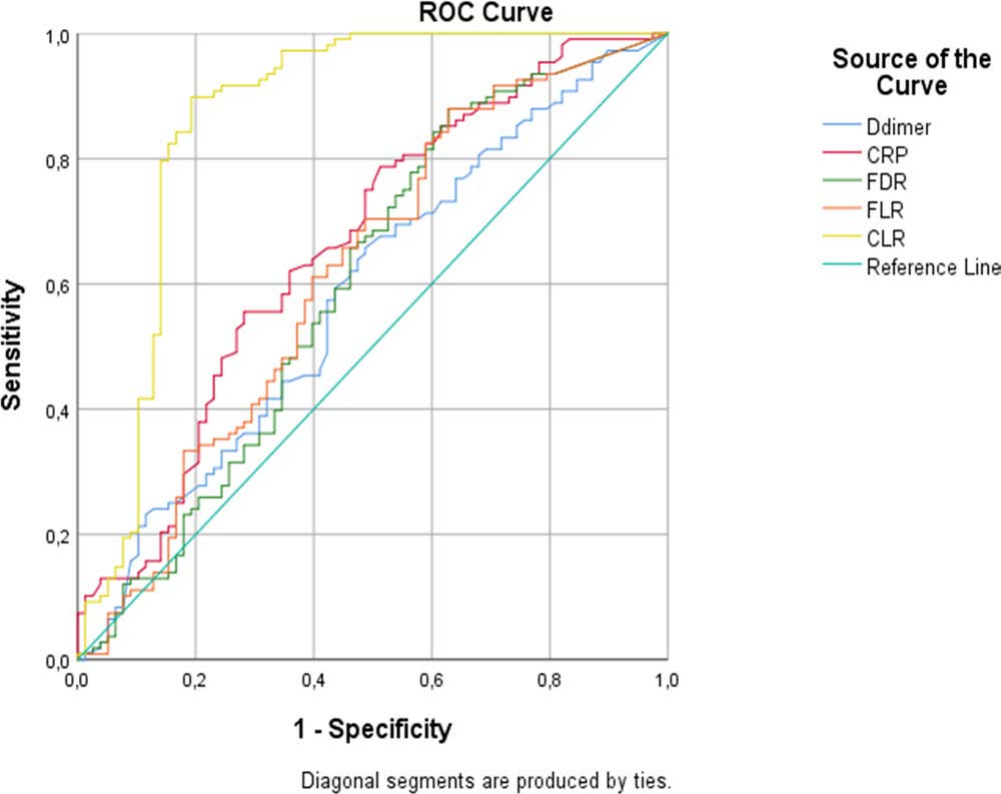
The ROC curve of D-dimer, C-reactive protein, ferritin D-dimer ratio (FDR), ferritin lymphocyte ratio (FLR), and C-reactive protein lymphocyte ratio (CLR) for discriminating between Coronavirus disease-2019 (COVID-19) patients with and without acute pulmonary embolism.

The median age turned out to be 61 ± 13 in the no-PE group, whereas 60 ± 18 in the PE group (Table 1). There was no statistical difference between the age of the groups (*P* = .6). Although the gender distribution in the no-PE group was 46 (43.8%) women and 59 (56.2%) men, it was 53 (41.7%) women and 74 (58.3%) men in the PE group. Eosinophil and lymphocytes remained lower, whereas D-dimer, CRP, FDR, FLR, and CLR levels were higher in the PE group (Table 1). Patients with PE had a longer hospital stay and more severe disease and death rates. Shortness of breath and tiredness was more common in the PE group. Most patients had segmental and/or subsegmental pulmonary embolism only 2 patients who had massive pulmonary embolism were excluded due to co-existing malignancies. The PE group also had lower SpO2 levels at admission. Optimal cutoff values calculated by the ROC analysis and the ROC curves are presented in Figure 2. When PE was compared to those no-PE the areas under the curve (AUC) of D-dimer, CRP, FDR, FLR and CLR were found as 0.58 (*P* = .035), 0.66 (*P* = .001), 0.60 (0.002), 0.61 (0.006), and 0.86 (*P* = .001), respectively (Table 2). The correlation analysis is shown in Table 3. Ferritin, CRP, FLR, and CLR were positively correlated with D-dimer whereas a negative correlation detected between platelet count neutrophil ratio and D-Dimer.

**Table 1:** Demographic data and laboratory findings of Coronavirus disease-2019 (COVID-19) patients with and without pulmonary embolism.

Variable	No-PE n = 105	PE n = 127	*P* value
Female	46 (43.8%)	53 (41.7%)	.712
Age (yr)	61 ± 13	60 ± 18	.621
White blood cell count 10^3^/μL	7.78 (1.7–18.7)	8.9 (1.35–39)	.032*
Neutrophil 10^3^/μL	5.11 (0.8–16)	6.3 (1.2–24)	.038*
Lymphocyte 10^3^/μL	1.38 (0.23–4.12)	1.12 (0.11–3.56)	.391
Eosinophil 10^3^/μL	0.14 (0–4)	0.02 (0–0.3)	.042*
Platelets 10^3^/μL	293 (112–755)	266 (30–652)	.061
D-dimer FEU/mL	2.4 (0.2–20)	4.1 (0.2–19)	.037*
Ferritin ng/mL	218 (45–1124)	403 (65–1323)	.008*
C-reactive protein mg/L	18 (0.3–258)	50 (8–552)	.042*
Ferritin D-dimer ratio	45 (5–612)	77 (3–655)	.017*
Ferritin C-reactive protein ratio	11 (5–325)	9 (6–430)	.121
Ferritin lymphocyte ratio	97 (9–543)	222 (8–1207)	.007*
CLR	12 (0.31–252)	56 (16–330)	.001*
Ferritin neutrophil ratio	39 (1.36–352)	51 (1.54–552)	.172
Platelet neutrophil ratio	50 (14.3–165)	45 (6.7–153)	.027*
Hospital stay (d)	7 (0–24)	8 (0–36)	.023*
Severe disease	20 (19%)	41 (32%)	.022*
Death	5 (4.8%)	19 (14.8%)	.012*
SpO2 (%)	94 (75–98)	92 (65–98)	.022*
Cough	28 (26.7%)	39 (30.5%)	.571
Shortness of breath	49 (46.7%)	86 (67.2%)	.002*
Fever	20 (19%)	14 (10.9%)	.083
Tiredness	15 (14.3%)	37 (28.9)	.008*
Joint pain	16 (15.2%)	28 (21.9%)	.195
COPD	5 (4.8%)	9 (7%)	.465
Asthma	7 (6.7%)	13 (10.2%)	.345
Diabetes mellitus	18 (17.1%)	22 (17.2%)	.951
Hypertension	36 (34.3%)	42 (32.8%)	.863
Coronary artery disease	14 (13.3%)	23 (18%)	.372

CLR = C-reactive protein lymphocyte ratio, COPD = chronic obstructive pulmonary disease, SpO2 = the percent saturation of oxygen in the blood.

* *P* < .05 statistically significant.

**Table 2:** ROC analysis of Coronavirus disease-2019 (COVID-19) patients with or without acute pulmonary embolism.

Variable	AUC (95%CI)	Cutoff	Sensitivity %	Specificity %	*P*
D-dimer	0.58	>3.8	58	59	.035*
CRP	0.66	>33	62	60	.001*
FDR	0.60	>58	59	58	.002*
FLR	0.61	>158	61	60	.006*
CLR	0.86	>81	83	83	.001*

AUC = area under the ROC curve, CLR = C-reactive protein lymphocyte ratio, CRP = C-reactive protein, FDR = ferritin D-dimer ratio, FLR = ferritin lymphocyte ratio, WBC = white blood cell count.

* *P* < .05 statistically significant.

**Table 3:** Spearman correlations between laboratory findings of Coronavirus disease-2019 (COVID-19) patients with and without acute pulmonary embolism.

		CRP	Ferritin	D-dimer	FDR	FLR	CLR	PNR
CRP	r	1	0.330*	0.28*	0.192*	0.207*	0.543*	−0.220*
	p		0.001	0.01	0.008	0.004	0.001	0.002
Ferritin	r	0.330*	1	0.175*	0.552*	0.734*	0.300*	−0.267*
	p	0.001		0.021	0.001	0.001	0.001	0.001
D-dimer	r	0.280*	0.175*	1	−0.287*	0.303*	0.187*	−0.222*
	p	0.01	0.021		0.001	0.001	0.01	0.002
FDR	r	0.192*	0.552*	−0.287*	1	0.679*	0.138	−0.048
	p	0.008	0.001	0.001		0.001	0.059	0.517
FLR	r	0.207*	0.734*	0.303*	0.679*	1	0.210*	−0.243*
	p	0.004	0.001	0.001	0.001		0.003	0.001
CLR	r	0.543*	0.300*	0.187*	0.138	0.210*	1	−0.212*
	p	0.001	0.001	0.01	0.059	0.003		0.003
PNR	r	−0.220*	−0.267*	−0.222*	−0.048	−0.243*	−0.212*	1
	p	0.002	0.001	0.002	0.517	0.001	0.003	

CLR = C-reactive protein lymphocyte ratio, CRP = C-reactive protein, FDR = ferritin D-dimer ratio, FLR = ferritin lymphocyte ratio, PNR = platelets neutrophil ratio.

* *P* < .05 statistically significant.

A multivariable model was developed to predict mortality risk, and odds ratios (OR) for statistically significant results are depicted in Table 4. Patients with higher white blood cell count (WBC) had an adjusted multivariable OR of 1.36 (*P* = .04). Patients with high ferritin, CRP, CLR and FLR had an increased risk of mortality at 1.2 (0.017), 1.3 (*P* = .022), 1.22 (*P* = .039), and 1.31 (*P* = .012), respectively.

**Table 4:** Logistic regression analysis of factors predicting risk of mortality.

Variable	OR	*P* value
WBC	1.36 (1.02–1.41)	.040
Ferritin	1.2 (1.13–1.38)	.017
CRP	1.3 (0.99–1.34)	.022
FLR	1.31 (1.03–1.44)	.012
CLR	1.22 (0.99–2.01)	.033
Age	1.07 (0.99–2.15)	.068

Data in parentheses are 95% confidence intervals.

CLR = C-reactive protein lymphocyte ratio, CRP = C-reactive protein, FLR = ferritin lymphocyte ratio, OR = odds ratio lymphocyte ratio, WBC = white blood cell count.

## 4. Discussion

Limited information is available about the co-existence of PE and COVID-19, which is an important and sometimes life-threatening condition. In the present study, some laboratory and clinical findings were different between COVID-19 patients with and without PE.

Pulmonary embolism and COVID-19 are diseases where patients usually suffer from shortness of breath. There are no definite distinguishing symptoms between the 2 conditions. Fever and cough were more frequent complaints among COVID-19 patients.^[7]^ Yet symptom frequency in the co-existence of PE and COVID-19 is not well studied, and more information is needed in this field. In the present study, tiredness and shortness of breath were more common symptoms in the PE group. The SpO2 level is undoubtedly a vital parameter that indicates well-being. In the present study, SpO2 levels at admission were lower in the PE group.

Both PE and COVID-19 are sometimes life-threatening conditions; recent reports show PE does not increase the mortality rate in COVID-19 patients.^[8,9]^ Unlike previous studies, the mortality rate was higher in the PE group. The study discrepancy might be due to patient characteristics or heterogeneity of COVID-19. As severe disease rates were higher in the PE group, it unclear if the PE embolism is a result or the reason for the severe disease.

Evidence suggests that the WBC is associated with adverse outcomes in patients with cardiovascular diseases via vascular plugging, direct injury to myocytes, and the coronary endothelium.^[10]^ Yet their role is uncertain in COVID-19 patients with PE. Previously Venetz et al reported WBC count was an independent predictor of short-term mortality and hospital readmission in patients with PE.^[11]^ In a study, Galland et al analyzed 88 patients who tested positive for SARS-CoV-2 and reported WBC ≥ 12.0 G/L independently associated with the PE diagnosis.^[12]^ In accordance with previous studies in the present study, high levels of WBC were associated with an increased mortality risk in COVID-19 patients with PTE. Eosinophils have been shown to have various functions like immunoregulation and antiviral activity, but their role in COVID-19 is not well known. In a study, Xie et al reported that COVID-19 patients had a decrease in circulating eosinophil counts, which are significantly more common than other pneumonia patients.^[13]^ The present study has demonstrated that circulating eosinophil counts decrease in the PE group (*P* = .042). In the light of obtained results and previous studies, as a common condition, investigating reasons for eosinopenia may help deal with COVID-19.

One of the most challenging situations in PE is thrombocytopenia. In patients with COVID-19, some patients develop thrombocytopenia, a severe pro-inflammatory state associated with a unique coagulopathy and procoagulant endothelial phenotype. The difficulty is choosing the appropriate anticoagulant while thrombocytopenia is on the other side. A previous study reported myocardial injury among COVID-19 patients with lower platelet counts increased mortality.^[14]^ In the present study, no difference was detected between platelet levels. The discrepancy between studies may be due to the heterogeneity of COVID-19, the patient characteristics, or the effects of PE.

D-dimer is a fibrin degradation product and is usually elevated in thrombotic events. Previously in the early phase of COVID-19 infection, high D-dimer levels have been reported^[15]^ and have been associated with a bad prognosis in patients with severe COVID-19.^[16]^ Klok et al reported an increased risk of venous thromboembolism in ICU patients with COVID-19.^[4]^ In the present study, D-dimer levels were higher in the PE group. The cutoff value was determined to predict a PE as 3.8 Feu/mL (AUC: 0.58, *P* = .035). No statistically significant results were found regarding mortality risk.

High ferritin levels have been associated with poor prognosis in patients with COVID-19.^[17]^ In the present study, ferritin levels were higher in the PE group, which were associated with increased mortality risk. CRP is an important inflammatory marker in which high levels have been associated with a need for mechanical ventilation and a bad prognosis in patients with COVID-19.^[18]^ In the present study, CRP levels were higher in the PE group and were positively correlated with D-dimer levels.

Researchers have been investigating ratios such as neutrophil/lymphocyte, platelet/lymphocyte, and monocyte/lymphocyte ratios in the diagnosis and prognosis of some inflammatory conditions.^[19]^ Previously Celik et al reported both NLR and PLRs higher in PE patients than controls as inflammatory markers in non-COVID patients.^[20]^ In a study, Seyit et al reported high NLR and PLR in patients who tested positive for SARS-CoV-2 compared to controls.^[6]^ A recent study reported that elevated NLR is significantly associated with the severity of COVID-19.^[5]^ Ferritin and CRP are essential biomarkers in patients with COVID-19, and high levels have been associated with poor prognosis.^[16]^ In the present study, we hypothesized that the ratios of biomarkers such as CRP and ferritin could help evaluate the diagnosis and prognosis of COVID-19 with PE. FDR, FLR, and CLR were higher in the PE group. C-reactive protein and lymphocytes both are routinely studied in most patients with COVID-19. C-reactive protein, lymphocytes, and CLR have been investigated in the diagnosis and prognosis of COVID-19 patients but there is a lack of information on CLR in co-existence of COVID-19 and PTE. In a previous study, Demirkol et al reported poor outcomes in COVID-19 patients with high CLR levels.^[21]^ In another study, Xiao et al reported elevated CLR was an independent risk factor for poor short-term clinical outcomes of COVID-19 patients.^[22]^ To the best of our knowledge, there are no previous studies investigating the importance of CLR in the diagnosis and prognosis of COVID-19 patients with PTE. In the present study, CLR had an AUC of 0.86 with 83 % sensitivity and specificity. High CLR was associated with increased mortality risk (OR: 1.22, *P* = .033). Despite the high sensitivity and specificity of ROC analysis, CLR had a weak correlation with D-dimer.

## 5. Limitations

This study has some limitations; even though all laboratory parameters belong to the time before using an in-hospital treatment, comorbid diseases or medications used for these diseases such as coronary artery disease, hypertension, and diabetes mellitus may have affected blood parameters. In addition to being a retrospective study, there was a difficulty in accessing some information due to the high number of patients who applied to the emergency services, pulmonary diseases, and COVID-19 clinics during the data collection period. Data that were not at a satisfactory level or missing were not included in the study. As severe disease rates were higher in the PE group, it unclear if the PE embolism is a result or the reason for the severe disease.

## 6. Conclusion

In conclusion, elevated CLR is highly predictive of PE and is associated with increased mortality risk in patients with COVID-19. Patients complaining of shortness of breath and tiredness accompanied by lower SpO2 levels and with a CLR level above 81 should be investigated for PE.

## Author contributions

**Conceptualization:** Ibrahim Koc, Deniz Olgun, Sevda Unalli Ozmen.

**Data curation:** Ibrahim Koc, Deniz Olgun, Sevda Unalli Ozmen, Sertan Bulut.

**Formal analysis:** Ibrahim Koc, Deniz Olgun.

**Funding acquisition:** Ibrahim Koc, Sevda Unalli Ozmen.

**Investigation:** Ibrahim Koc, Sevda Unalli Ozmen, Sertan Bulut.

**Methodology:** Ibrahim Koc, Sertan Bulut.

**Project administration:** Ibrahim Koc, Sevda Unalli Ozmen, Sertan Bulut.

**Resources:** Ibrahim Koc.

**Software:** Ibrahim Koc, Deniz Olgun, Sevda Unalli Ozmen.

**Supervision:** Ibrahim Koc, Deniz Olgun, Sevda Unalli Ozmen.

**Validation:** Ibrahim Koc, Sertan Bulut.

**Visualization:** Ibrahim Koc, Sertan Bulut.

**Writing – original draft:** Ibrahim Koc, Sertan Bulut.

**Writing – review & editing:** Ibrahim Koc.
